# Protocol for preparation of primary alveolar epithelial type I cells from mouse lungs

**DOI:** 10.1016/j.xpro.2024.103484

**Published:** 2024-12-12

**Authors:** Lulu Huang, Amy Tang, Bojing Shao

**Affiliations:** 1 Laboratory of Vascular Inflammation and Thrombosis Research, Lindsley F. Kimball Research Institute, New York Blood Center, New York, NY 10065, USA

**Keywords:** cell biology, cell culture, cell isolation, cell separation/fractionation, mass cytometry

## Abstract

Currently, the only approach for obtaining alveolar epithelial type I (AT1) cells is through the differentiation of cultured AT2 cells, which has several limitations. Here, we present a protocol to prepare AT1 cells directly from mouse lungs. We detail the steps for dissociating epithelial cells from the alveoli, followed by the purification of AT1 cells. Additionally, we describe how to assess the purity of AT1 cells using immunofluorescence staining and flow cytometry, along with approaches for verifying their functions.

## Highlights

• Steps for harvesting lungs and dissociating epithelial cells from alveoli
• Protocols for culturing isolated AT1 cells *in vitro*
• Assays for characterizing prepared primary AT1 cells

## Abstract (editor highlights)

Publisher’s note: Undertaking any experimental protocol requires adherence to local institutional guidelines for laboratory safety and ethics.

## Graphical abstract

**Figure:**
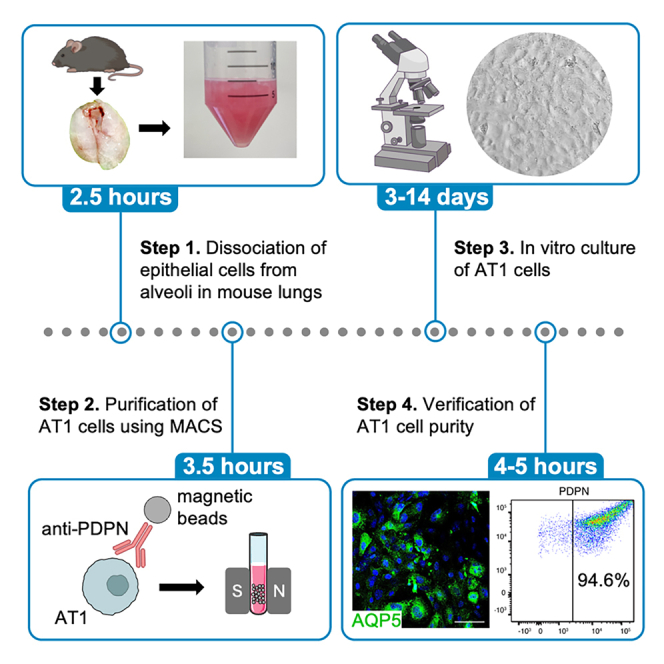

## Before you begin

Alveolar epithelial type I (AT1) cells cover 95% of the alveolar inner surface and serve the leading role in gas exchange. With retained cellular plasticity, AT1 cells also contribute to alveolar regeneration after lung injury.^1^ Currently, AT1 cells used in most studies are differentiated from cultured alveolar epithelial type II (AT2) cells of lungs. Yet, AT1 cells derived *in vitro* are highly heterogeneous in cellular identity and functions, which increases the experimental variability.^2^ In addition, although mouse models are preferred in genetic editing applications, mouse AT1 cells are rarely used in studies.^3^

The protocol below describes the detailed steps for directly preparing mouse primary AT1s with a high cell yield. We developed a purification procedure using the positive-selection magnetic-activated cell sorting (MACS) to isolate AT1 cells from the digested mouse alveoli. Then, with brief *in vitro* cell culturing, one to three million of AT1 cells with a decent purity (89.47 ± 5.08%, mean ± SD) were acquired per mouse. Thus, this protocol is practical in preparing mouse primary AT1 cells to study pulmonary diseases. In addition, comparing directly isolated AT1 cells with AT1 cells derived from AT2 cells would provide more opportunities to learn the mechanism for AT2 cell differentiation and the function of AT1 cells under various pathophysiological conditions.

### Institutional permissions

All laboratory mice were maintained under standard husbandry and housing conditions approved by the New York Blood Center Institutional Animal Care and Use Committee (IACUC). All animals were handled in accordance with the NIH and Association for Assessment and Accreditation of Laboratory Animal Care (AAALAC) guidelines for humane care and use of laboratory animals.

## Key resources table

**Table:** 

REAGENT or RESOURCE	SOURCE	IDENTIFIER
**Antibodies**		

Biotinylated Syrian hamster anti-mouse podoplanin (PDPN) monoclonal antibody (eBio8.1.1 (8.1.1)) (1:50 dilution)	Thermo Fisher Scientific	Cat# 14-5381-82; RRID: AB_1210505
Rabbit anti-mouse aquaporin 5 (AQP5) (1:40 dilution)	Alomone Labs	Cat# AQP-005; RRID: AB_2039736
Rabbit anti-mouse RAGE (1:100 dilution)	Boster	Cat# A03438; RRID: AB_3081636
Rabbit IgG (1:20 dilution)	Santa Cruz	Cat# Sc-3888; RRID: AB_737196
CY3, goat anti-Syrian hamster IgG (H + L) (1:75 dilution)	Jackson ImmunoResearch Laboratories	Cat# 107-165-142; RRID: AB_2337464
Alexa Fluor 488, donkey anti-rabbit IgG (H + L) (1:100 dilution)	Thermo Fisher Scientific	Cat# A21206; RRID: AB_2535792

**Chemicals, peptides, and recombinant proteins**		

Dispase	STEMCELL	Cat# 7913
DNase I	Sigma-Aldrich	Cat# DN25
PBS buffer powder	VWR	Cat# 76371-734
Hanks’ balanced salt solution, 1× without calcium, magnesium, and phenol red (HBSS)	Corning Inc.	Cat# 21-022-CV
Fetal bovine serum	Thermo Fisher Scientific	Cat# 16-000-044
RPMI 1640	Sigma-Aldrich	Cat# R8758
Dulbecco’s modified Eagle’s medium and Ham’s F-12	Sigma-Aldrich	Cat# D8062
Penicillin-streptomycin solution, 100×	Corning Inc.	Cat# 30-002-CI
Magnetic streptavidin particles	BD Biosciences	Cat# 557812
Agarose	Promega	Cat# V3121
DAPI	BioLegend	Cat# 422801
Red blood cell lysing solution	BD Biosciences	Cat# 555899
16% paraformaldehyde	Electron Microscopy Sciences	Cat# 15710
BSA	Sigma-Aldrich	Cat# A3733
Donkey serum	Sigma-Aldrich	Cat# D9663
Goat serum	Thermo Fisher Scientific	Cat# 16210064
10% Triton X-100	Sigma-Aldrich	Cat# 93443
Mounting medium	Sigma-Aldrich	Cat# F4680
Tween 20	Sigma-Aldrich	Cat# P1379
Cytometric bead array (CBA) mouse IL-6 flex set	BD Biosciences	Cat# 558301

**Experimental models: Organisms/strains**		

C57BL/6J (males and females, 4–6 months old)	The Jackson Laboratory	Cat# 000664

**Software and algorithms**		

Fiji	National Institutes of Health	https://fiji.sc

**Other**		

70 μm nylon cell strainer	Falcon	Cat# 352350
SURFLO winged infusion set	Terumo	Cat# SV∗27EL
Shaking incubator	VWR	Cat# 76407-112
Water bath	VWR	Cat# 76308-832
Variable speed rotator with tube holders	Benchmark Scientific	Cat# R2024
Tissue culture plate 24 well	Corning Inc.	Cat# 09-761-146
10 mL syringe	BD	Cat# 302995
23G needle	BD	Cat# 305145
3/0 silk braided suture thread	Kits of Medicine	Cat# 7625938343475
15 mL conical sterile polypropylene centrifuge tubes	Thermo Fisher Scientific	Cat# 339650
50 mL conical sterile polypropylene centrifuge tubes	Thermo Fisher Scientific	Cat# 339652
5 mL polystyrene tubes	Greiner Bio-One	Cat# 115101
Magnetic rack	BD Biosciences	Cat# 552311

## Materials and equipment

**Table:** Digestion medium

Reagent	Final concentration	Amount
RPMI 1640 medium	N/A	3.6 mL
DNase I	100 μg/mL	0.4 mL

***Note:*** Prepare fresh digestion medium for every experiment.

**Table:** Suspension medium

Reagent	Final concentration	Amount
RPMI 1640 medium	N/A	4.5 mL
Fetal Bovine Serum	50%	5 mL
DNase I	100 μg/mL	1 mL

***Note:*** Prepare fresh suspension medium for every experiment.

**Table:** FACS buffer

Reagent	Final concentration	Amount
PBS 1×	N/A	98 mL
Fetal Bovine Serum	2%	2 mL

***Note:*** FACS buffer can be prepared in advance and can be stored for up to 2 months at 4°C.

**Table:** AT1 Cell medium

Reagent	Final concentration	Amount
Dulbecco’s modified Eagle’s medium and Ham’s F-12	N/A	79 mL
Fetal Bovine Serum	20%	20 mL
Penicillin/Streptomycin 100×	1%	1 mL

***Note:*** AT1 Cell medium can be prepared in advance and can be stored for up to 1 month at 4°C.

**Table:** Blocking buffer

Reagent	Final concentration	Amount
PBS 1×	N/A	7.85 mL
20% BSA	3%	1.5 mL
Donkey serum	3%	0.3 mL
Goat serum	3%	0.3 mL
10% Triton X-100	0.05%	0.05 mL

***Note:*** Blocking buffer can be prepared in advance and stored for up to 2 weeks at 4°C.

### Primary antibody buffer

• Add 6 mL PBS 1× buffer to a 15 mL tube.
• Add 3 mL blocking buffer.
• Mix thoroughly and store on ice.

***Note:*** Prepare fresh primary antibody buffer for every experiment.

### Permeabilization buffer

• Add 9.95 mL PBS 1× buffer to a 15 mL tube.
• Add 50 μL Tween 20.
• Mix thoroughly and store at room temperature.

***Note:*** Blocking buffer can be prepared in advance and stored for up to 2 weeks.

### 1% agarose solution

• Weigh 0.2 g agarose and transfer to a bottle.
• Add 20 mL PBS 1× buffer.
• Boil in microwave to dissolve; the agarose should appear transparent.
• Store at 45°C water bath for up to 10 h.

## Step-by-step method details

### Dissociate epithelial cells from alveoli in mouse lungs

**Timing: 2.5 h**

This step describes the protocol of harvesting the lungs and dissociating epithelial cells from the alveoli.

**Figure 1:**
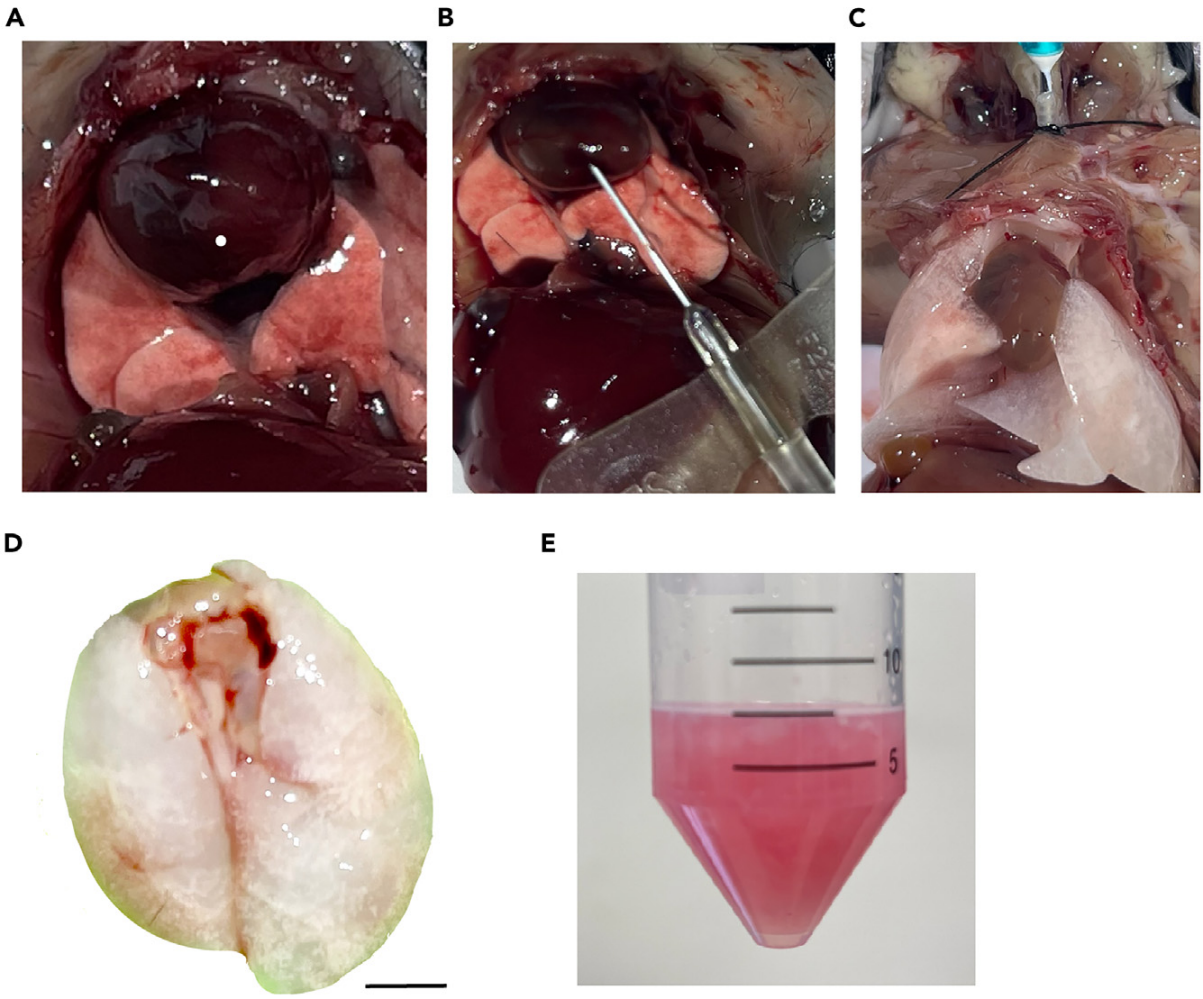
Dissection of mouse lungs and dissociation of alveolar cells (A) The mouse lung before perfusion. (B) Image illustrates perfusing the lung through right ventricle with a SURFLO winged infusion set. (C) The inflated lung with dispase and agarose. (D) After digestion of dispase, lung lobes are separated. Scale bar: 5 mm. (E) Example of lung dissociation at indicated incubation time with enzymes.

1. Sacrifice two adult mice after application of isoflurane inhalation followed by cervical dislocation.
2. Make an abdominal incision and fully expose the diaphragm.
  a. Incise the diaphragm to expose the sternum (Figure 1A).
  b. Make a cut 1 cm in length on the liver to flush out the circulating blood. (This is an alternative method to flush blood out of the vasculature, which will prevent cell injury by coagulation activation and reduce contamination of blood cells during isolation of AT1 cells).
  c. Perfuse the lungs using a 10 mL syringe and SURFLO winged infusion set with a 27G needle to inject sterile HBSS through the right ventricle (Figure 1B). Generally, 10 mL HBSS is sufficient to perfuse an adult mouse and perfusion should be completed around 1 min.
3. Ligate and cannulate the trachea.
  a. Make an incision to the neck.
  b. Remove the thyroid and trachealis muscle completely.
  c. Nick the trachea, cannulate it with a 23-gauge needle and secure the needle with the 3/0 suture thread.
  d. Inject 1 mL dispase and following 1 mL melted 1% agarose through the needle (Figure 1C).
  e. Immediately cover the lungs with ice for 5 min.

**CRITICAL:** It’s important to let the agarose solidify and seal the upper airways quickly, which prevents bronchial epithelial cells from dissociating from the upper airways.

4. Remove the needle once lungs are taken out of ice and dissect the mice.
  a. Obtain the lungs without the trachea (Figure 1D).
  b. Transfer the lungs to a new 50 mL tube containing 0.5 mL dispase.
  c. Incubate for 1 h on a shaker, 37°C, 180 RPM.
5. Add 4 mL digestion medium and finely mince the lungs.
  a. Cut 2–3 mm from the tip of a 1 mL pipette tip.
  b. Pipette the tissue pieces up and down over 20 times, to further dissociate the cells.
  c. Incubate for 20 min on a shaker, 37°C, 200 RPM (Figure 1E).
6. Add 10 mL suspension medium of RPMI 1640 containing 50% FBS and 100 μg/mL DNase I.
  a. Vortex the suspension gently for 5 s, to allow cell aggregate dissociation.
  b. Incubate on ice for 5 min.
  c. Filter the suspension through a 70 μm nylon cell strainer.
  d. Transfer the single cell suspension to a new 50 mL centrifuge tube.
  e. Centrifuge at 500 *g*, 4°C for 10 min.
  f. Discard the supernatant.
7. Resuspend cell pellets in 3 mL 1× RBC lysis buffer.
  a. Incubate for 5 min at room temperature (RT).
  b. Add 10 mL PBS containing 40% FBS to stop the reaction.
  c. Centrifuge at 500 *g*, 4°C for 10 min.
  d. Discard the supernatant.
8. Resuspend cell pellets in 250 μL of FACS buffer.

***Note:*** Cell counts at this step should be around 3–6 × 10^7^ per mouse.

### Purify alveolar epithelial cells type I using positive-selection MACS

**Timing: 3.5 h**

This step describes the protocol of purifying AT1 cells using AT1 marker PDPN and magnetic-activated cell sorting assay.

9. Add 5 μL of biotinylated Syrian hamster anti-mouse PDPN to cell suspension from step 8.
  a. Incubate for 1 h on a rotator at 4°C.
  b. Centrifuge at 500 *g*, 4°C for 6 min.
  c. Discard the supernatant.
  d. Resuspend in 1 mL FACS buffer, repeat step 9b.
  e. Discard the supernatant.
10. Resuspend cell pellets in 500 μL FACS buffer.
  a. Add 30 μL of streptavidin conjugated magnetic particles.
    **CRITICAL:** Vortex the beads thoroughly before use.
  b. Incubate for 1 h on a rotator at 4°C.
11. Transfer the mixture to a round-bottom 5 mL polystyrene tube (1^st^).
  a. Add another 500 μL FACS buffer.
  b. Let the tube stand on a magnetic rack for 8 min, 4°C.
    ***Note:*** Other magnetic racks (such as purchased from Miltenyi) or column-based magnetic racks also work for this step.
  c. AT1 cells will bind to the magnetic beads and remain on the inner wall of the tube next to the magnetic rack.
    ***Note:*** A brown line on the inner wall containing beads should be seen at this step.
  d. Carefully remove the supernatant, followed by detaching magnetic beads from the inner wall of the tube with 1 mL FACS buffer. Then transfer the bead-containing buffer from the 1^st^ tube to the 2^nd^ tube, repeat selection as in step 11b.
    **CRITICAL:** Hold the tube still during transfer of cells to obtain the maximum number of bead-bound cells.
  e. Repeat step 11d and transfer magnetic beads in the 2^nd^ tube to the 3^rd^ tube, then repeat selection as in step 11b.
  f. Repeat step 11d and transfer magnetic beads in the 3^rd^ tube to the 4^th^ tube, then repeat selection as in step 11b. After removing the supernatant, detach AT1 cells from the wall of the 4^th^ tube with 0.5 mL culture medium.
12. Resuspend AT1 cells from the fourth tubes with AT1 culture medium.
  a. Dilute AT1 suspension to an appropriate density (e.g., 2.5 × 10^5^/mL).
  b. Add 1 mL mixture to each well in a 24-well plate.

***Note:*** The yield of AT1 cells is approximately 2%–3% of the total cells used for purification.

13. Culture AT1 cells in 37°C incubator with 5% CO_2_.
  a. Change the medium every 3–5 days.
    **CRITICAL:** AT1 cells could spread and reach confluence but are barely reproductive. Thus, there is no need for passage.
  b. Culture for no more than 2 weeks.

### Characterization of AT1 cells purity through immunofluorescence staining

**Timing: 5 h**

This step describes the protocol of characterizing the purity of AT1 cells with immunofluorescence staining.

14. Discard the cell supernatants in 35 mm glass-bottom petri dish, wash once with PBS buffer.
15. Fix cells with fresh 2% paraformaldehyde, RT, 10 min.
  a. Remove 2% PFA.
  b. Wash twice with PBS, 5 min each.
16. Block cells with blocking buffer, RT, 1 h.
  a. Remove blocking buffer.
17. Dilute rabbit anti-mouse AQP5 and rabbit IgG in primary antibody buffer to a final concentration of 10 μg/mL.
  a. Incubate AT1 cells with different primary antibody dilutions, RT, 1 h.
  b. Remove primary antibody dilutions.
  c. Wash AT1 cells three times with PBS, 5 min each.
18. Dilute AF488 donkey anti-rabbit secondary antibody in PBS to a final concentration of 10 μg/mL.
  a. Incubate AT1 cells with secondary antibody dilution, RT, 1 h, avoiding the light.
  b. Remove primary antibody dilutions.
  c. Wash AT1s three times with PBS, 5 min each.
19. Dilute DAPI in PBS to a final concentration of 10 μg/mL.
  a. Incubate AT1 cells with DAPI, RT, 15 min, avoiding the light.
  b. Remove DAPI dilution.
  c. Wash once with PBS, 5 min.
20. Mount AT1 cells with mounting medium and capture images using confocal laser scanning microscope.

### Characterization of AT1 cells purity through flow cytometry

**Timing: 4 h**

This optional step describes the protocol of using flow cytometry to characterize the purity of AT1 cells.

21. Harvest cultured AT1 cells from 24-well plate.
  a. Remove the supernatant wash once with PBS.
  b. Add Trypsin-EDTA, incubate for 2 min at 37°C.
  c. Add AT1 medium to stop the reaction and pipette the cells gently.
  d. Transfer the cell suspension to a 15 mL tube, centrifuge at 200 *g*, 5 min, 4°C.
  e. Discard the supernatant.
22. Resuspend cell pellets with 1 mL fresh 2% paraformaldehyde, RT, 10 min.
  a. Centrifuge at 200 *g*, 5 min, 4°C.
  b. Discard the supernatant.
  c. Resuspend in FACS buffer, centrifuge at 200 *g*, 5 min, 4°C.
23. Permeabilize cells by incubating with Tween 20.
  a. Centrifuge at 200 *g*, 5 min, 4°C.
  b. Discard the supernatant.
  c. Resuspend the pellets in 1 mL permeabilization buffer, incubate at RT for 10 min.
24. Wash the cells twice.
  a. Centrifuge at 200 *g*, 5 min, 4°C.
  b. Discard the supernatant.
  c. Resuspend in FACS buffer, centrifuge at 200 *g*, 5 min, 4°C.
25. Block cells with the blocking buffer.
  a. Centrifuge at 200 *g*, 5 min, 4°C.
  b. Discard the supernatant.
  c. Resuspend in blocking buffer.
  d. Incubate at RT for 30 min in the dark.
  e. Centrifuge at 200 *g*, 5 min, 4°C.
  f. Discard the supernatant.
26. Dilute rabbit anti-mouse RAGE or Syrian hamster anti-mouse podoplanin and rabbit or Syrian hamster IgG in FACS buffer to a final concentration of 10 μg/mL.
  a. Resuspend pellets with primary antibodies, incubate for 1 h, RT, avoiding the light.
  b. Add 1 mL FACS buffer to each tube, centrifuge at 200 *g*, 5 min, 4°C.
  c. Discard the supernatant.
27. Dilute AF488 conjugated donkey anti-rabbit secondary antibody and CY3 conjugated goat anti-Syrian hamster secondary antibody in PBS 1× to a final concentration of 20 μg/mL.
  a. Resuspend pellets with secondary antibody dilutions, incubate for 30 min, RT, avoiding the light.
  b. Add 400 μL PBS to each tube.
28. Proceed to detection in the flow cytometer.

### Functional validation of AT1 cells

**Timing: 1 day 4 h**

This step describes the protocol of validating the function of AT1 cells using anti-RAGE antibody and flow cytometry.

29. Discard the cell supernatants in 24-well plate at Day 5.
30. Incubate AT1s with anti-RAGE antibody.
  a. Mix anti-RAGE antibody with DMEM at the concentration of 10 μg/mL.
  b. Add 200 μL mixture to each well.
  c. Add 200 μL DMEM to wells as the control group.
  d. Incubate for 24 h in 37°C incubator with 5% CO2.
31. Collect the supernatants.
  a. Centrifuge at 1000 *g*, 4°C for 10 min, to remove remaining cells.
32. Perform Cytometric Bead Array following the manufacturer’s instruction.
33. Proceed to detection in the flow cytometer.

## Expected outcomes

**Figure 2:**
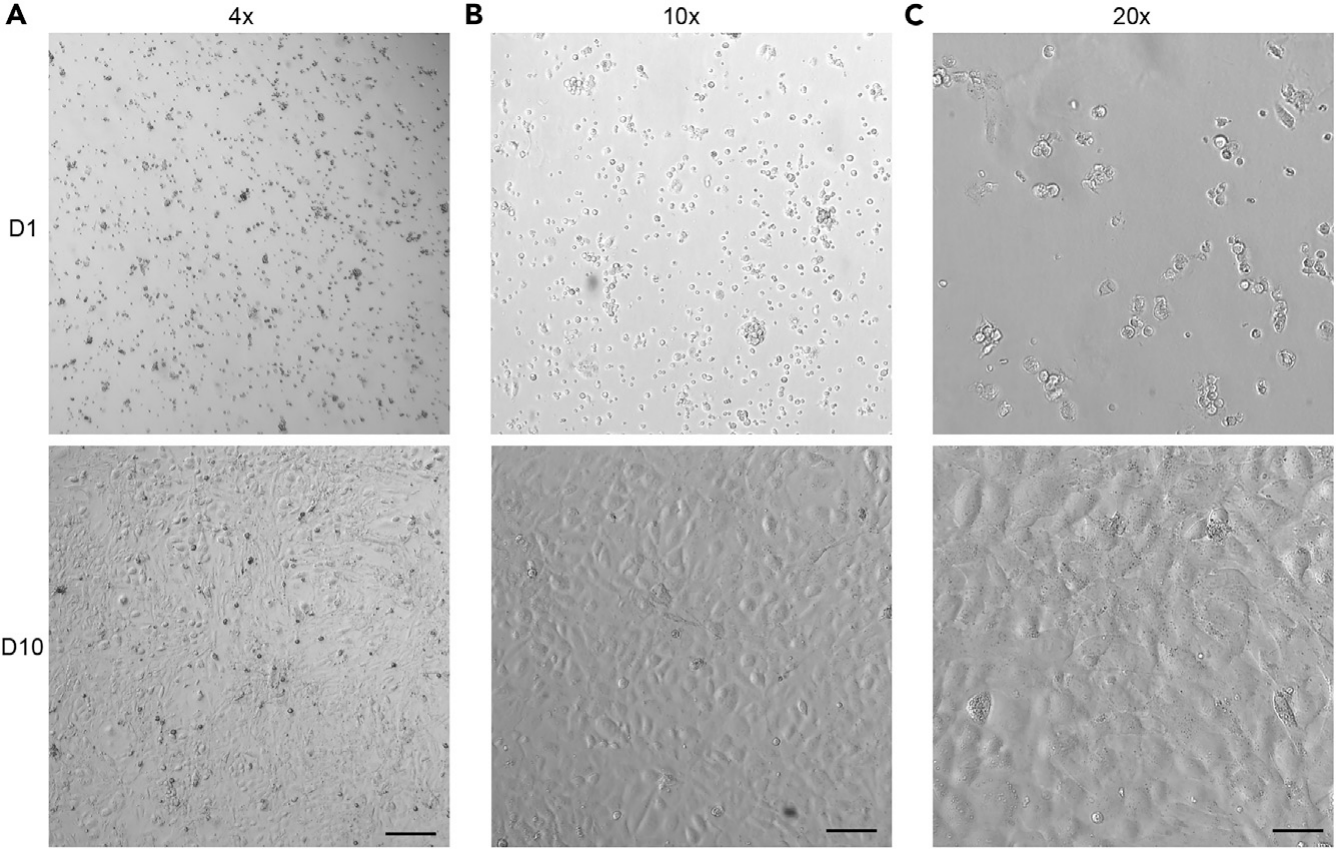
Purified AT1 cells cultured in 24-well plate Images showing cultured AT1 cells at Day 1 (D1) and Day 10 (D10), with 4× (A), 10× (B) and 20× (C) magnification. AT1 cells are seeded into 24-well plate at a density of 2.5 × 10^5^ cells per well. The cells become confluent at D10 with the cobblestone morphology. Scale bar: 250 μm (A), 100 μm (B) and 50 μm (C).

This step-by-step protocol provides detailed information for the isolation and purification of primary alveolar type 1 (AT1) cells and their culture. After purifying AT1 cells, we seed them into a 24-well plate and observe the cells grow and spread (Figure 2). AT1 cells start to show typical cobblestone morphology after adhering to the plate and become confluent at D10.

**Figure 3:**
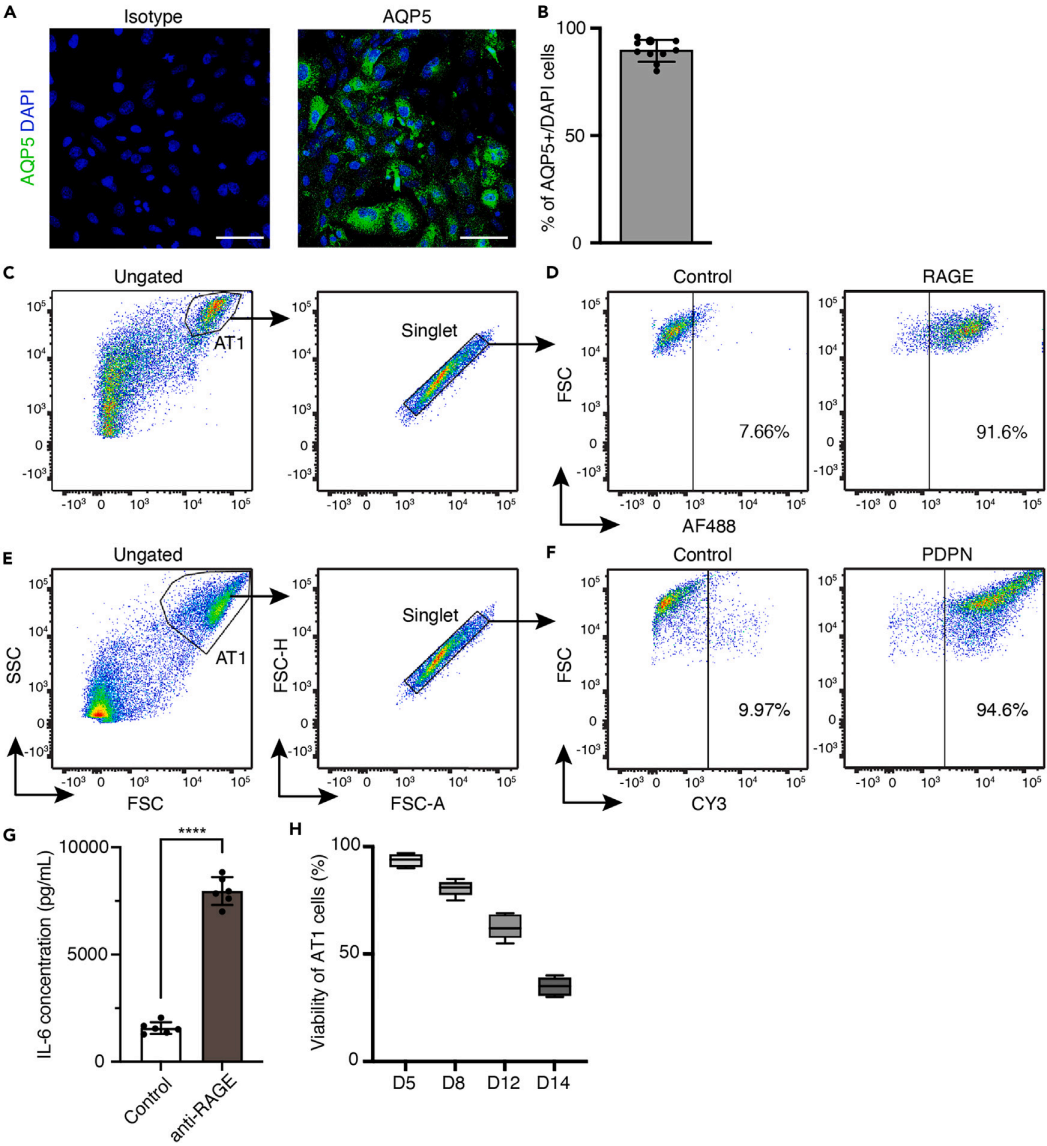
Characterization of purified AT1 cells (A) Immunofluorescence confocal microscope images of AT1 cells cultured for 5 days. Aquaporin5 (AQP5) is a marker of AT1 cells. Scale bar: 50 μm. (B) Quantification of AQP5-positive cells. (C) Dot plot of purified cells cultured for 5 days stained with anti-RAGE, AT1 cells were gated as the black polygon and singlets were gated as the diagonal. FSC, forward scatter; SSC, side scatter; FSC-H, forward scatter height; FSC-A, forward scatter area. (D) Dot plot of gated single AT1 cells with AF488 fluorescence intensity as X axis. (E) Dot plot of purified cells cultured for 5 days stained with anti-PDPN, AT1 cells were gated as the black polygon and singlets were gated as the diagonal. (F) Dot plot of gated single AT1 cells with CY3 fluorescence intensity as X axis. (G) Interleukin-6 concentration in the supernatants of AT1 cells incubated for 24 h with or without anti-RAGE antibody. (H) Viability of AT1 cells at different time points as day 5, 8, 12, and 14 (D5, D8, D12, D14). Data represent mean ± SD. ∗∗∗∗, p < 0.0001.

To assess the efficiency of this protocol, we use confocal microscope to capture images of AT1 cells stained with anti-AQP5, then quantify AQP5-positive cells, and get a high purify at 89.47 ± 5% (Figures 3A and 3B). Additionally, we stain cells with anti-RAGE and anti-PDPN, then analyze the cells using flow cytometry (Figures 3C–3F), which also indicates the high purity of purified AT1 cells. To further validate the identity of AT1 cells, we incubated AT1 cells with anti-RAGE antibody and measured the level of Interleukin-6 (IL-6), as one of the functional properties of *in vivo* AT1 cells under inflammatory stimulation (Figure 3G).

## Limitations

The above protocol can be applied to primary alveolar epithelial type I cells from mouse lungs. It decreases the heterogeneity often seen within of AT2-differentiated AT1 cells. However, the lack of self-renewal ability results in a limited time frame (∼2 weeks) for studies related to AT1 cells (Figure 3H).

## Troubleshooting

### Problem 1

Purified AT1 cell count is low.

### Potential solution

Perfuse lungs well that will allow a better exposure of alveolar cells to enzymes. Adjust the incubation time with dispase and DNase I, mince the lung thoroughly (e.g., cutting lung lobes to small pieces followed by homogenizing with tissue grinders) or increase the dosage of anti-PDPN antibodies and magnetic beads.

### Problem 2

Purified AT1 cells have low viability.

### Potential solution

Speed up the epithelial cell dissociation process and keep the cells on ice as much as possible. Gently pipette to resuspend cells, no vertex, every time after pelleting cells with centrifugation.

### Problem 3

Seeded AT1 cells cannot reach confluency.

### Potential solution

Optimize the seeding density, for example, we seed 2.5 × 10^5^ AT1 cells to each well of a 24-well plate. In addition, a short spin of 400 *g* × 5 min at room temperature for the plate will increase the seeding of cells reaching confluency.

## Resource availability

### Lead contact

Further information and requests for resources and reagents should be directed to and will be fulfilled by the lead contact, Dr. Bojing Shao (bshao@nybc.org).

### Technical contact

Technical questions on executing this protocol should be directed to and will be answered by the technical contact, Dr. Lulu Huang (lhuang@nybc.org).

### Materials availability

This study did not generate new unique reagents.

### Data and code availability

This study did not generate/analyze datasets or code.

## Acknowledgments

The study was supported by grants from the National Institutes of Health (NIAID, 1R21AI171525 to B.S.). We thank the flow cytometry core of the Lindsley F. Kimball Research Institute of the New York Blood Center for its technical support.

## Author contributions

L.H. and A.T. performed experiments. B.S. conceived the study and interpreted data. L.H. and B.S. wrote the manuscript with input from all authors.

## Declaration of interests

The authors declare no competing interests.

## References

[1] Flodby P., Kim Y.H., Beard L.L., Gao D., Ji Y., Kage H., Liebler J.M., Minoo P., Kim K.J., Borok Z., Crandall E.D. (2016). Knockout Mice Reveal a Major Role for Alveolar Epithelial Type I Cells in Alveolar Fluid Clearance. Am. J. Respir. Cell Mol. Biol..

[2] Chen Q., Liu Y. (2021). Isolation and culture of mouse alveolar type II cells to study type II to type I cell differentiation. STAR Protoc..

[3] Gonzalez R.F., Dobbs L.G. (2013). Isolation and culture of alveolar epithelial Type I and Type II cells from rat lungs. Methods Mol. Biol..

